# Violence and psychosis: Clinical evidences from an Early Intervention Program

**DOI:** 10.1192/j.eurpsy.2023.340

**Published:** 2023-07-19

**Authors:** O. Mentxaka, M. Recio-Barbero, E. Arana-Arri, R. Segarra

**Affiliations:** 1 Department of Psychiatry, Cruces University Hospital; 2 Early Stages of Psychosis Group, Biocruces Bizkaia Health Research Institute, Barakaldo; 3 Department of Psychiatry, University of the Basque Country, Leioa; 4 Scientific Coordinator, Biocruces Bizkaia Health Research Institute, Barakaldo; 5 Centro de Investigación Biomédica en Red de Salud Mental, CIBERSAM, Leioa, Spain

## Abstract

**Introduction:**

Psychotic disorders are frequently linked to a public perception of dangerousness and propensity to engage in violent acts. Despite efforts to demystify these disorders, the evidence on the relationship between violence and psychotic disorders is mixed. Together with media coverage of violent crime associating violence with the occurrence of a mental disorder, such a situation has contributed to the social stigmatisation of people with severe mental disorders and the consequent discrimination that this scenario entails. Despite efforts to demystify such disorders, the association between violent behaviour and psychosis remains unclear.

**Objectives:**

This study aims to explore the incidence and main clinical characteristics associated to violent offences recorded in a cohort of patients presenting a First-Episode Psychosis (FEP).

**Methods:**

Patients presenting with an affective or non-affective first psychotic episode were recruited from the First Episode Psychosis Intervention Program (CRUPEP) cohort between 2009 and 2016. The main clinical variables were collected, including medical-forensic records of patients registered at the Basque Institute of Forensic Medicine (BIFM), to retrieve any violent acts in which patients with FEP were involved, either as victims or as offenders.

**Results:**

Overall, 79.5% (n=182) of CRUPEP patients had no violent record of crime or offence recorded in the BIFM. Annual crime rates for the 2009–2016 period show a decreasing trend in both the general population (IRR=0.981 (95%CI=0.978–0.983) p<0.001) and in patients with FEPs (IRR=0.019 (95%CI=0.012–0.028) p<0.001); this pattern is more pronounced the FEP group. Victimisation accounted for the vast majority of reported incidents; nevertheless, patients who have committed violent offences were mostly involved in intrafamily violence

**Conclusions:**

Patients with FEP were not involved in a higher number of crime rates than the general population. The types of violent acts committed by FEP patients were heterogeneous, with extreme violence being particularly uncommon.

**Disclosure of Interest:**

None Declared

